# Preoperative short-course radiotherapy versus combined radiochemotherapy in locally advanced rectal cancer: a multi-centre prospectively randomised study of the Berlin Cancer Society

**DOI:** 10.1186/1471-2407-9-50

**Published:** 2009-02-06

**Authors:** Robert Siegel, Susen Burock, Klaus-Dieter Wernecke, Albrecht Kretzschmar, Manfred Dietel, Volker Loy, Stephan Koswig, Volker Budach, Peter M Schlag

**Affiliations:** 1 Department of Surgery and Surgical Oncology, Charité – Universitätsmedizin Berlin, Berlin, Germany; 2 Institute of Medical Informatics and Biometry, Charité – Universitätsmedizin Berlin, Berlin, Germany; 3 Department of Haematology and Oncology, Helios Kliniken, Berlin, Germany; 4 Institute of Pathology, Charité – Universitätsmedizin Berlin, Berlin, Germany; 5 Institute of Pathology, Vivantes Hospital, Berlin, Germany; 6 Department of Radiotherapy, Helios Kliniken, Bad Saarow, Germany; 7 Department of Surgery and Surgical Oncology, Charité Comprehensive Cancer Center, Charité – Universitätsmedizin Berlin, Berlin, Germany

## Abstract

**Background:**

The additional use of radiotherapy has changed the treatment of locally advanced rectal cancer (LARC) dramatically. But a major achievement has been the development of total mesorectal excision (TME) as a surgical standard and the recognition that the surgeon is the predominant prognostic factor. The benefit of preoperative hypofractionated radiotherapy (SCRT; five fractions each of 5 Gy), initially established by the Swedish Rectal Cancer Trial, has been demonstrated in conjunction with TME by the Dutch Colorectal Cancer Group. The concept of combined neoadjuvant radiochemotherapy (conventional radiation of about 50 Gy with chemotherapy) has not been compared over surgery alone with TME. However, the German Rectal Cancer Study Group recently demonstrated that preoperative radiochemotherapy (RCT) was better than postoperative radiochemotherapy in terms of local control.

**Methods and design:**

Patients with histological proven rectal cancer staged T2N+ or T3 are randomized to receive either SCRT (25 Gy in five fractions of 5 Gy) plus TME-surgery within 5 days or RCT (50.4 Gy in 28 fractions of 1.8 Gy, continuous infusion 5-fluorouracil) plus TME-surgery 4–6 weeks later. All patients receive adjuvant chemotherapy (12 weeks continuous infusional 5-FU) and are followed up for 5 years. TME-quality is independently documented by the surgeon and the pathologist. Hypothesis of the study is that RCT is superior to SCRT in terms of local recurrence after five years. Secondary endpoints are overall survival, disease-free survival, complete resection rate (R0 resection), rate of sphincter saving resection, acute and late toxicity (radiation related side effects), and quality of life (including long term bowel function).

**Discussion:**

Similar long-term survival, local control and late morbidity have been reported for both concepts of preoperative therapy in non-comparative studies. In addition to other ongoing (and recently published) comparative trials we include a larger number of patients for adequate power, apply quality-controlled TME and try to avoid the adjuvant treatment bias by mandatory adjuvant chemotherapy in both groups. Further more, stratification of the initially planned surgical procedure and sphincter-preservation will generate valid evidence whether RCT will allow a less aggressive (sphincter saving) surgical approach.

## Background

Colorectal cancer (CRC) is the second most frequent malignant disease in western countries. In Germany, more than 70,000 new cases occur yearly and approximately 40% are located in the rectum [1]. Despite major efforts in prevention/screening and significant improvements in surgical, radiation and medical therapy CRC is still one of the leading causes of cancer mortality.

Besides survival, a major problem in locally advanced rectal cancer (LARC) is the thread of local recurrence, not only because of the limited therapeutic options but especially because of the impaired quality of life due to the intense pain and uncontrolled soiling (caused by faecal incontinence and/or mucus/blood from the tumour).

The last decade has brought advances in multimodality treatment of LARC. After the endorsement of postoperative adjuvant radiochemotherapy by a National Cancer Institute Consensus Conference in 1990 [2], several randomized studies reported lower rates of local failure with preoperative radiotherapy than with surgery alone [3,4]. Furthermore, the Swedish Rectal Cancer Trial, evaluating preoperative short-course radiotherapy (SCRT), found an advantage in overall survival compared to surgery alone [5]. The recently published results from the CAO/ARO/AIO-94 trial report that preoperative combined radiochemotherapy (RCT) is superior to postoperative RCT in terms of outcome [6]. A major achievement in the treatment of LARC has been the development of total mesorectal excision (TME) as a surgical standard [7]. So far, the only study evaluating preoperative therapy together with a documented quality controlled TME-surgery is the Dutch Colorectal Cancer Group study. SCRT with TME was superior to TME alone in terms of local recurrence, whereas overall survival was similar in both groups [4]. The EORTC 22921 trial, already started in the late nineties of the last century and published recently, showed a similar reduction in local recurrence whether 5FU/leucovorin chemotherapy was given with pre-operative radiotherapy, after preoperative radiotherapy plus surgery, or both [8].

In Europe, there is still much debate about the two different approaches to preoperative therapy – SCRT and "long-course" RCT. Although having undoubted weakness, a recently published study of the Polish Colorectal Study Group comparing the two approaches reported no superiority for RCT in terms of either overall or local control [9]. Even in the U.S., there is now increasing demand in considering the use of SCRT in order to better stratify for adjuvant therapy, because of the low probability of downsizing and therefore "unchanged" initial TN-staging [10].

Our study, the Berlin Rectal Cancer Trial (BRCT) was planned in 2003 and enrolled the first patient in 2004. After initiation of the polish trial and the BRCT in Europe, a comparative trial very similar to the BRCT was conducted in Australia. This TROG/AGITG trial recently closed recruitment after enrolling some three hundred patients. In addition to the recently published polish trial we include a larger number of patients for adequate power, applied quality-controlled TME and tried to avoid the adjuvant treatment bias by mandatory adjuvant chemotherapy in both groups. Further more, stratification of the initially planned surgical procedure and sphincter-preservation will generate valid evidence whether RCT will allow a less aggressive (sphincter saving) surgical approach.

Hypothesis of our study is that RCT is superior to SCRT in terms of local recurrence after five years.

## Methods and design

### Study design

**Figure 1:**
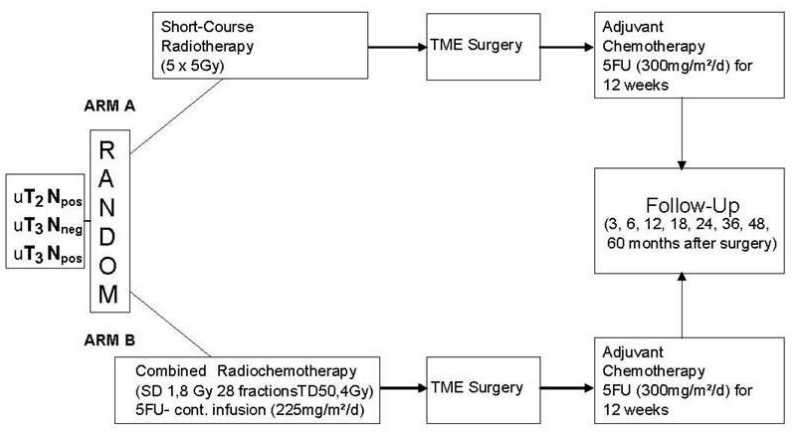
**Treatment algorithm**.

The study is a two-arm prospective randomised multicentre trial. The treatment algorithm is depicted in Figure 1.

### Study objectives

The study compares the value of the two most common preoperative therapy regimens in LARC in terms of local recurrence. The primary outcome measure is local recurrence after a follow up of five years. The secondary outcome measures are overall survival, disease-free survival and quality of life (including long term bowel function) after a median follow-up of five years. Further more, secondary endpoints are the complete resection rate (R0 resection) and the rate of sphincter saving resection, both analysed after surgery of the last patient recruited. Acute and late toxicity (radiation related side effects) will be observed after a median follow-up of five years.

### Trial organization

BRCT has been designed by the Department of Surgery and Surgical Oncology (Charité, Campus Berlin-Buch) in cooperation with the Department of Radiotherapy (Charité, Campus Berlin-Buch and Campus Mitte), the Department of Haematology, Oncology and Tumorimmunology (Charité, Campus Berlin-Buch) and the Institute of Medical Informatics and Biometry (Charité), Berlin, Germany. The trial is an investigator initiated trial. The BRCT is supported by the Berlin Cancer Society (Berliner Krebsgesellschaft e.V.).

### Coordination

The trial is coordinated by the clinical trial office of the Charité Comprehensive Cancer Center, Charité, Berlin. The clinical trial office is responsible for overall trial management, trial registration (EudraCT Number 2004-001606-27; Current Controlled Trials ISRCTN56463377), database management, quality assurance including monitoring, reporting and for the scientific program of all trial related meetings supported by the Berliner Krebsgesellschaft e.V..

### Investigators

Patients are recruited in the following centres in Germany: Robert-Rössle-Klinik Charité, Berlin; Helios Klinikum Emil-von-Behring, Berlin; Oskar-Ziethen-Krankenhaus, Berlin; Virchow-Klinkum Charité, Berlin; DRK-Klinikum Westend, Berlin; Krankenhaus Düsseldorf-Gerresheim, Düsseldorf; DRK-Krankenhaus Luckenwalde, Luckenwalde; Schlossparkklinik, Berlin; Helios Klinikum Schwerin, Schwerin; St.-Joseph-Krankenhaus, Berlin; DRK-Kliniken Köpenick, Berlin; DRK-Klinikum Westend, Berlin; Dominikus Krankenhaus, Berlin; Vivantes Humboldt Klinikum, Berlin; Universitätsklinikum Münster, Münster; Vivantes Klinikum Neukölln, Berlin; Kreiskrankenhaus Prignitz, Prignitz; Evangelisches Krankenhaus Königin Elisabeth Herzberge, Berlin; Krankenhaus Waldfriede, Berlin; St. Hedwig Klinikum, Berlin; Gemeinschaftskrankenhaus Havelhöhe, Berlin; Klinikum Ludwigsburg, Ludwigsburg; Universitätsklinikum Düsseldorf, Düsseldorf; Benjamin-Franklin-Klinikum Charité, Berlin; Martin Luther Krankenhaus, Berlin; Klinikum Ernst von Bergmann, Potsdam; Unfallkrankenhaus Marzahn, Berlin; Caritas Klinik Maria Heimsuchung, Berlin; Universitätsklinikum Essen, Essen; Klinikum Sindelfingen-Böblingen, Böblingen; Evangelisch-Freikirchliches-Krankenhaus Bernau, Bernau; Helios Klinikum Berlin, Berlin; Vivantes Klinikum am Urban, Berlin; Universitätsklinikum Leipzig, Leipzig; Vivantes Klinikum Spandau, Berlin; Henriettenstiftung Hannover, Hannover; Krankenhaus Siloah Hannover, Hannover; Helios Klinikum Wuppertal, Wuppertal; Krankenhaus Hedwigshöhe, Berlin.

### On-site monitoring

During recruitment of patients monitoring on site is performed according to good clinical practice (GCP) guidelines. The data management will be performed by the clinical trial office of the Charité Comprehensive Cancer Center, Charité, Berlin.

### Ethics, informed consent and safety

The final protocol was approved by the ethics committee of the Charité, Berlin, Germany (ref: AA3/03/38). It is certified and recommended by the German Cancer Society (Deutsche Krebsgesellschaft e.V., "Gütesiegel A", 29th September 2003). This study complies with the Helsinki Declaration in its recent version, the Medical Association's professional code of conduct, the principles of Good Clinical Practice (GCP) guidelines and the Federal Data Protection Act. The trial will also be carried out in keeping with local legal and regulatory requirements. The medical secrecy and the Federal Data Protection Act will be followed. Written informed consent is obtained from each patient in oral and written form before inclusion in the trial and the nature, scope, and possible consequences of the trial have been explained by a physician. The investigator will not undertake any measures specifically required only for the clinical trial until valid consent has been obtained.

### Statistics

Local recurrence after five years follow up has been chosen as primary endpoint. Survival in the regarded treatment arms will be graphically demonstrated by using Kaplan-Meier estimates. Univariate Log-Rank tests as well as multivariate Cox' regression analyses will be conducted for the analysis of primary and secondary endpoints. The alteration of hazard ratios in time will be modelled [11]. Proving a two-sided hypothesis of differences between the treatment arms, the calculation of the sample size of the trial was based on expected local recurrence rates of 12% in the SCRT arm compared with 7% in the RCT arm, resulting in altogether 760 patients (Log-Rank-test, base time: 5 years, accrual time: 6 years, follow-up: 5 years, proportion loss to follow-up: 10% per arm, error of the 1^st ^kind: α = 5% two-sided, power = 80%). An interim analysis is planned after five years study time with an error of the 1^st ^kind = α_int _= 0.5% [12].

### Randomisation and stratification

Randomisation is done centrally at the clinical trial office of the Charité Comprehensive Cancer Center, Charité, Berlin. During randomisation patients are stratified by centre, tumour location (below 3 cm, between 3 and 8 cm, or between 8 and 12 cm above dentate line), TN-stage (uT2N+, T3N0, T3N+), open vs. laparoscopic surgery, and the type of resection (abdominoperineal resection vs. low anterior resection) according to the surgeons evaluation prior to preoperative therapy. Minimization has been used to ensure a balanced distribution with respect to arms and strata [13].

### Patient selection

In order to be included in the trial, patients are required to be aged 18 years or over and to have

1. Karnofsky Index 80% or better

2. Histological proved diagnosis of rectal cancer (adeno- or mucinous carcinoma)

3. Primary rectal cancer:

3.1. Maximum 12 cm above dentate line (upper limit)

3.2. Staged T2N+ or T3N0 or T3N+ (by endorectal ultrasound or Computed Tomography [CT]/Magnetic Resonance Imaging [MRI] scan)

4. No evidence of metastatic disease as determined by chest X-ray and abdominal ultrasound (or CT-scan of chest and abdomen or other investigations such as Positron Emission Tomography [PET] scan or biopsy if required)

5. Adequate bone marrow function with platelets more than 100 × 10^9/l and neutrophils more than 2.0 × 10^9/l

6. Creatinine clearance more than 50 ml/min

7. Serum bilirubin less than 2.0 × Upper Limit of institutional Normal range (ULN)

8. Written informed consent is obtained prior to commencement of trial treatment.

Exclusion criteria are:

1. Rectal cancer other than adeno- or mucinous carcinoma

2. Previous or concurrent malignancies, with the exception of adequately treated basal cell carcinoma of the skin or in situ carcinoma of the cervix

3. Patients with locally advanced inoperable disease, such as T4-tumour

4. Presence of metastatic disease or recurrent rectal tumour

5. Any previous chemotherapy or radiotherapy, and any investigational treatment for rectal cancer

6. Concurrent uncontrolled medical conditions

7. Pregnancy or breast feeding

8. Clinically significant (i.e. active) cardiac disease (e.g. congestive heart failure, symptomatic coronary artery disease) or myocardial infarction within the last six months

9. Stenotic tumour which can not be passed by the colonoscope and pre-operative need of diverting stoma

10. Evidence of hereditary colorectal cancer (Hereditary Non-Polyposis Colorectal Cancer [HNPCC] and Familial Adenomatous Polyposis [FAP])

11. Medical or psychiatric conditions that compromise the patient's ability to give informed consent

### Preoperative therapy

Radiotherapy is performed according to the standardized protocol with CT-based 3D-conformal treatment planning. The clinical target volume includes the primary tumour, the mesorectal tissue including perirectal and presacral nodes, and internal iliac lymph nodes [14]. The reference dose is defined in accordance to the International Commission on Radiation Units and Measurement report 50/63. All patients are irradiated in a prone position on a belly board with a CT-planned three-field technique with megavoltage photons ≥ 6 MV. An individual field shielding is obligatory by using a multileaf collimator. SCRT consists of single doses of 5.0 Gy in five fractions within one week up to a total dose of 25 Gy. For RCT, standard fractions of 1.8 Gy/d are given 5 times a week up to a total dose of 50.4 Gy; concomitant chemotherapy consists of continuous 5-FU-infusion 225 mg per square meter per day.

Radiotherapy-related toxicities will be assessed using the NCI Common Toxicity Criteria (CTC). Toxicity will be evaluated weekly during the therapy and at follow-up 3 months, 6 months, 1 year, 2 years and 5 years after treatment by standard forms (LENT SOMA).

### Surgery and histopathological analysis

Surgical resection has to be done within 5 days after SCRT or 5–6 weeks after the completion of RCT. The standard surgical procedure is total mesorectal excision (TME), for tumours located 10 cm above the anal verge a partial mesorectal excision (PME) is feasible; anterior resection, intersphincteric resection or abdominoperineal resection is done according to clinical staging and patient's preference. Quality (completeness) of TME has do be evaluated and documented by every surgeon at the resected specimen (by injection of methylene blue into the arteria rectalis superior).

The surgical specimens are processed and evaluated by standardized pathology and including UICC TNM categories and staging groups, number of examined and involved lymph nodes and the status of resection margins. In addition, the CRM (circumferential resection margin) and the TME-quality have to be documented by the pathologist according to the British Medical Research Council [15]. Histopathological response is deduced from tumour regression (TRG). TRG is evaluated according the 5 point scoring system of Dworak [16].

### Adjuvant chemotherapy and follow-up

2–4 weeks after complete resection continuous 5-FU infusion therapy with 300 mg/m^2 ^BSA is applied for 12 weeks as far as there is no contraindication as serious impaired wound healing or anastomotic leakage. Toxicity will be evaluated during the chemotherapy and at follow-up.

Patients are included into a standardized follow-up program according to the guidelines of the Tumorzentrum Berlin. This includes follow-up visits at 3, 6, 12, 18 and 24 months after surgery, and then every year for a total of 5 years. Each visit includes physical examination, abdominal ultrasound, rectosigmoidoscopy and endorectal ultrasound (in case of sphincter saving surgery), CEA level as well as a chest X-ray every year. In addition, pelvic CT (or MRI) -scans are performed 3 months, 12 months, 24 and 36 months after surgery. Furthermore a complete colonoscopy is required 18 months, 36 months and 60 months post treatment.

Radiotherapy-related toxicities will be assessed during every visit using the LENT SOMA criteria.

Evaluation of quality of life is assessed by the EORTC QLQ D30 and QLQ D 38 questionnaire at time of randomisation, end of therapy, 3 and 6 months, 1 year, 3 years and 5 years after surgery.

## Discussion

Both preoperative short term radiotherapy (STRT) and neoadjuvant radiochemotherapy (RCT) provide increased local control of rectal cancer. With the present trial (with 476 patients already enrolled and an interim analysis to be performed at the end of 2008), we aim to definitely clarify the role of the different preoperative therapy regimen in rectal cancer concerning local control as well as long-term toxicities and overall survival. Especially together with the so far unreleased data from the recently closed TROG/AGITG trial with some more than 300 patients (and an almost identical study scheme) our trial will help to finally decide on the efficacy to increase local control, so further investigation can concentrate on improving survival by selecting and modifying adjuvant therapy regimen.

Metastasis free survival and overall survival are mainly influenced by adjuvant chemotherapy in high risk patients. Because of the fact that the histopathological staging after RCT seems not to be a useful indicator for adjuvant chemotherapy due to downstaging, adjuvant chemotherapy with 5-FU is performed in both groups independent of the histopathological staging to avoid an adjuvant treatment bias.

In case local control will be equally influenced by both therapy regimens, further follow-up with long-term toxicities and overall survival could help to determine the best protocol in rectal cancer treatment.

## Competing interests

The authors declare that they have no competing interests.

## Authors' contributions

RS and SB wrote the manuscript and are study coordinators. PMS and VB developed the study and wrote the initial study protocol. SK and AK supported the development of the study protocol. MD and VL are coordinating the histopathological analysis. KDW is responsible for the statistics. All authors read and approved the final manuscript.

## Pre-publication history

The pre-publication history for this paper can be accessed here:

http://www.biomedcentral.com/1471-2407/9/50/prepub
